# Non-Invasive Blood Pressure Estimation Enhanced by Capillary Refill Time Modulation of PPG Signals

**DOI:** 10.3390/s26010345

**Published:** 2026-01-05

**Authors:** Qianheng Yin, Yixiong Chen, Lan Lin, Dongdong Wang, Shen Sun

**Affiliations:** 1 College of Chemistry and Life Sciences, Beijing University of Technology, No. 100 Pingleyuan, Chaoyang District, Beijing 100124, China; 2 Beijing Science and Technology Project Manager Management Corporation Ltd., Beijing 100084, China; chenyixiong516@msn.com

**Keywords:** blood pressure, capillary refill time, photoplethysmography, machine learning, artificial intelligence

## Abstract

This study evaluates the impact of capillary refill time (CRT) modulation on photoplethysmography (PPG) signals for improved non-invasive continuous blood pressure (CBP) estimation. Data from 21 healthy participants were collected, applying a standardized 9 N pressure for 15 s to induce CRT during 6-min sessions. PPG signals were segmented into 252 paired 30-s intervals (CRT-modulated and standard). Three machine learning models—ResNetCNN, LSTM, and Transformer—were validated using leave-one-subject-out (LOSO) and non-LOSO methods. CRT modulation significantly enhanced accuracy across all models. ResNetCNN showed substantial improvements, reducing mean absolute error (MAE) by up to 35.6% and mean absolute percentage error (MAPE) by up to 40.6%. LSTM and Transformer models also achieved notable accuracy gains. All models met the Association for the Advancement of Medical Instrumentation (AAMI) criteria (mean error < 5 mmHg; standard deviation < 8 mmHg). The findings suggest CRT modulation’s strong potential to improve wearable CBP monitoring, especially in resource-limited settings.

## 1. Introduction

Continuous blood pressure (CBP) monitoring is essential for the effective management of cardiovascular disease (CVD), a leading global cause of mortality that accounts for approximately 17.9 million deaths annually [1,2]. Elevated blood pressure, a primary risk factor for CVD, necessitates frequent monitoring to reduces the risk of severe complications such as stroke and heart failure [3]. Traditional cuff-based methods (e.g., oscillometry) provide accurate intermittent measurements but are impractical for continuous, ambulatory monitoring due to their discomfort and bulky design [4,5,6]. Consequently, non-invasive alternatives have attracted considerable attention, with photoplethysmography (PPG) emerging as a promising optical technique due to its simplicity, cost-effectiveness, and seamless integration into wearable devices [7,8].

The field of PPG-based blood pressure estimation has evolved significantly over the past four decades, driven by advances in sensor technology, signal processing, and computational methodologies. In the 1980s, early research focused on pulse transit time (PTT), defined as the time delay between the electrocardiogram (ECG) R-wave and the PPG pulse peak, as a surrogate for blood pressure estimation [9,10]. Although PTT-based approaches showed promise, their reliance on simultaneous ECG measurements limited their applicability in standalone wearable devices.

By the early 2000s, advancements in PPG sensor design and signal processing facilitated the development of standalone PPG-based methods, which leverage morphological features—such as pulse amplitude, rise time, and waveform shape—combined with linear regression or machine learning models to estimate blood pressure directly [11,12]. The introduction of multi-wavelength PPG sensors (e.g., green, red, and infrared) marked a significant milestone, as these devices capture blood volume changes at varying tissue depths, enhancing the robustness of heart rate, oxygen saturation, and blood pressure monitoring [13,14].

In the past decade, the integration of deep learning techniques, including convolutional neural networks (CNNs), recurrent neural networks (RNNs), and transformer architectures, has advanced PPG-based CBP estimation by modeling complex, non-linear relationships between PPG signals and blood pressure [15,16,17,18]. Despite recent technological advances, several critical challenges remain in PPG-based blood pressure estimation. Physiological variability—resulting from individual differences in skin thickness, vascular compliance, and autonomic nervous system activity—introduces significant inconsistencies across subjects and conditions [19,20]. Additionally, motion artifacts, which are particularly prevalent during ambulatory monitoring, substantially degrade signal quality. The inherently non-linear and subject-specific relationship between PPG waveforms and blood pressure further complicates accurate estimation [21,22,23]. Moreover, existing methods often demonstrate reduced accuracy in heterogeneous populations and real-world environments, where external factors such as temperature, humidity, and inter-subject variability exacerbate measurement errors [24]. Most current approaches rely on static PPG features, including PTT and waveform morphology, which may inadequately capture the dynamic hemodynamic changes associated with blood pressure fluctuations—especially those induced by physiological stressors [25].

To address these limitations, we introduce CRT (Capillary Refill Time) modulation, a clinical indicator of peripheral perfusion, into PPG signals to supply dynamic, context-sensitive information. CRT is defined as the time taken for the skin’s color to return after blanching [6]. Under conditions of adequate blood volume, blood vessels in the skin’s proximal vascular bed remain naturally dilated, allowing significant blood flow. When substantial pressure is applied, blood is displaced towards the periphery, leading to skin pallor. Upon releasing the pressure, blood flow promptly resumes, reverting the skin to its normal perfused state. The duration of color restoration is referred to as CRT. A controlled pressure stimulus (9 N for 15 s) induces CRT, modulates microcirculatory dynamics, and produces time-varying hemodynamic features that complement static metrics such as PTT, thereby enhancing the physiological informativeness of PPG signals [26,27]. The selection of 9 N pressure was based on recent studies optimizing pressure for standardized CRT measurements, which recommend a range of 4.5–10.5 N to achieve stable and reliable CRT values, with pressures in this range ensuring consistent blanching and minimizing variability due to incomplete capillary emptying. This pressure level aligns with physiological mechanisms where sufficient force (around 9–10 N) is needed to fully expel blood from distal capillaries, allowing accurate assessment of refill dynamics without causing tissue damage, as higher pressures beyond 10.5 N may introduce inconsistencies [28]. The 15-s duration was selected based on established protocols for precise CRT assessment, as demonstrated in Ait-Oufella et al. (2014), where a 15-s compression time was applied to the index fingertip in septic shock patients to ensure adequate blanching, evidenced by the appearance of a thin white distal crescent under the nail [29]. This extended hold physiologically allows for complete blood displacement from the capillary bed, enabling reliable measurement of microcirculatory refill dynamics and reflecting peripheral perfusion status, which is adaptable to our study on healthy participants for standardized induction of CRT modulation. While vascular compliance varies with age, health status, and other factors, this fixed parameter provides a standardized starting point for healthy participants, with future work planned to explore adaptive pressures.

In this study, we evaluated the feasibility and effectiveness of CRT-modulated PPG for CBP estimation using three deep-learning architectures—ResNet-CNN, long short-term memory (LSTM), and Transformer models. The primary objective was to quantify the performance improvements attributable to CRT modulation within each architecture, thereby providing evidence of its potential applicability to scalable, wearable CBP monitoring, particularly in resource-constrained environments.

## 2. Methods

### 2.1. Study Protocol and Data Acquisition

This study was conducted from May to August 2024 in the Department of Biomedical Engineering, School of Chemistry and Life Sciences, Beijing University of Technology, and was reviewed and approved by the Ethics Committee of Beijing University of Technology (HS202507001). A cohort of 21 healthy volunteers (10 females, 11 males, aged 20–25 years) was recruited. As a pilot feasibility assessment, a cohort of 21 healthy volunteers (10 females, 11 males, aged 20–25 years) was recruited, consistent with initial exploratory studies in PPG-based blood pressure estimation where small sample sizes (e.g., 6–26 participants) are commonly used to demonstrate proof of concept and identify promising directions prior to larger-scale validations, despite limitations in generalizability and potential for higher uncertainty in deep learning models [30,31].

All participants were required to be free from any significant organ dysfunction or peripheral vascular disease. Demographic and clinical characteristics, including age, gender, height, weight, SBP, and DBP, are summarized in Table 1.

To maximize the utility of the dataset despite the constrained participant number, signals were segmented into 252 intervals per condition, incorporating demographic features to enhance model robustness and provide preliminary reference significance for CRT modulation’s potential in resource-limited settings.

**Figure 1:**
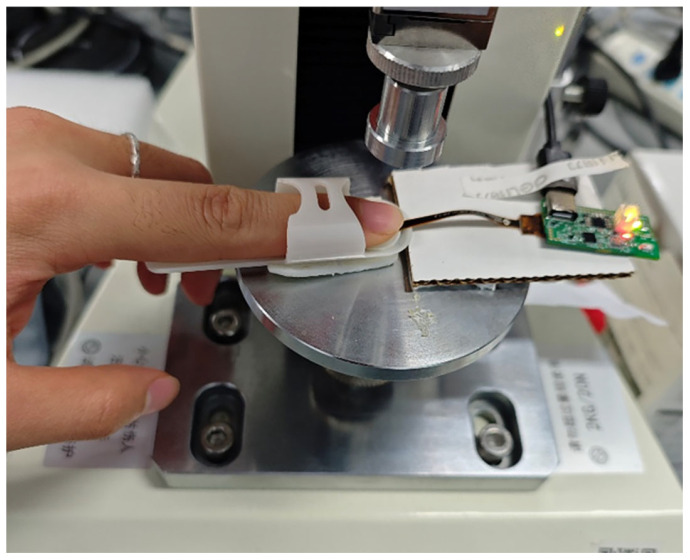
Experimental setup for signal acquisition. Photographs show the positioning of the participant’s finger during data collection, with the PPG sensor applied to the left index fingertip for CRT modulation and signal recording.

All measurements were conducted in a temperature- and humidity-controlled laboratory environment, with ambient temperature maintained at 22–24 °C and relative humidity at 30–50%. Prior to data acquisition, participants were seated and instructed to rest for 15 min to ensure hemodynamic stabilization and minimize motion-induced artifacts. The experimental setup is depicted in Figure 1. CRT modulation and PPG signal acquisition were performed on the left index fingertip using the MAXM86161 sensor (Mouser Electronics, Mansfield, TX, USA) module. This sensor features multi-wavelength light sources emitting at 660 nm (red), 880 nm (infrared), and 520 nm (green), integrated with a high-sensitivity photodiode and a low-noise analog front-end for precise optical signal detection [8,32,33].

PPG signals were sampled at 128 Hz, a frequency sufficient to capture the full range of physiological cardiac activity (0.5–3 Hz) as well as transient responses associated with CRT induction, without risk of aliasing [32,34]. Signal transmission was achieved via an I^2^C interface to the MAXM86161EVSYS1.0.0.0 software, and real-time data processing was conducted on a workstation equipped with an Intel^®^ Core™ i5-10300H processor. To ensure consistency in hemodynamic measurements, each participant’s forearm was positioned so that the fingertip was aligned with heart level throughout data collection [27].

The experimental protocol consisted of two 6-min recording sessions per participant, resulting in two distinct datasets: (1) a CRT-modulated PPG dataset, in which a controlled pressure of 9 N was applied to the left fingertip for 15 s at the 3rd and 5th minutes to induce microcirculatory changes, and (2) a baseline PPG dataset without CRT modulation. Each dataset was segmented into 30-s intervals, yielding a total of 252 data segments per condition (21 participants × 12 segments).

Reference systolic and diastolic blood pressure values were measured using a validated oscillometric device (Yuwell 670A, manufactured by Yuwell Medical Equipment Co., Ltd., Danyang, China) on the contralateral arm immediately after completion of each 6-min PPG recording session and served as ground-truth labels. This post-session timing was adopted to capture a stabilized hemodynamic state after participant acclimatization, thereby minimizing white coat hypertension that typically elevates pre-procedure readings due to anticipatory anxiety [35,36]. In rested healthy young adults, short-term BP variability over 6 min is negligible, rendering post-session measurement a reliable and widely accepted reference in cuffless BP validation studies.

**Figure 2:**
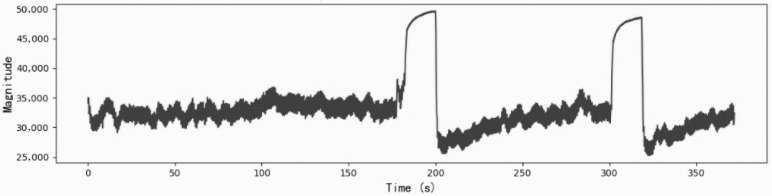
Representative PPG signal acquired during CRT modulation. The signal reflects microcirculatory responses to controlled fingertip pressure stimuli (9 N for 15 s), applied at the 3rd and 5th minutes of the recording session.

**Figure 3:**
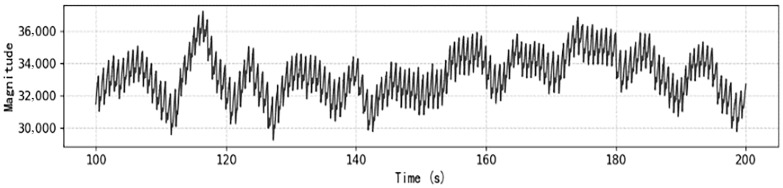
Representative baseline PPG signal without CRT modulation.

To account for physiological variability across individuals, demographic information—including gender, age, height, and weight—was collected and incorporated into the analysis. Representative examples of the raw CRT-modulated PPG signal and baseline PPG signal are presented in Figure 2 and Figure 3, respectively.

### 2.2. Initial Signal Preprocessing and Data Preparation

To enhance the quality of CRT-modulated photoplethysmography (PPG) signals while preserving both cardiac and microcirculatory characteristics, a structured pre-processing pipeline was developed. The pipeline consists of two stages: signal pre-processing and data preparation. These stages are designed to mitigate baseline drift, suppress high-frequency noise, and reduce inter-subject variability, while retaining CRT-induced hemodynamic features. This approach ensures the physiological integrity of the signals and provides a consistent and standardized input for effective training across all three deep learning models.

#### 2.2.1. Initial Signal Preprocessing

**Figure 4:**
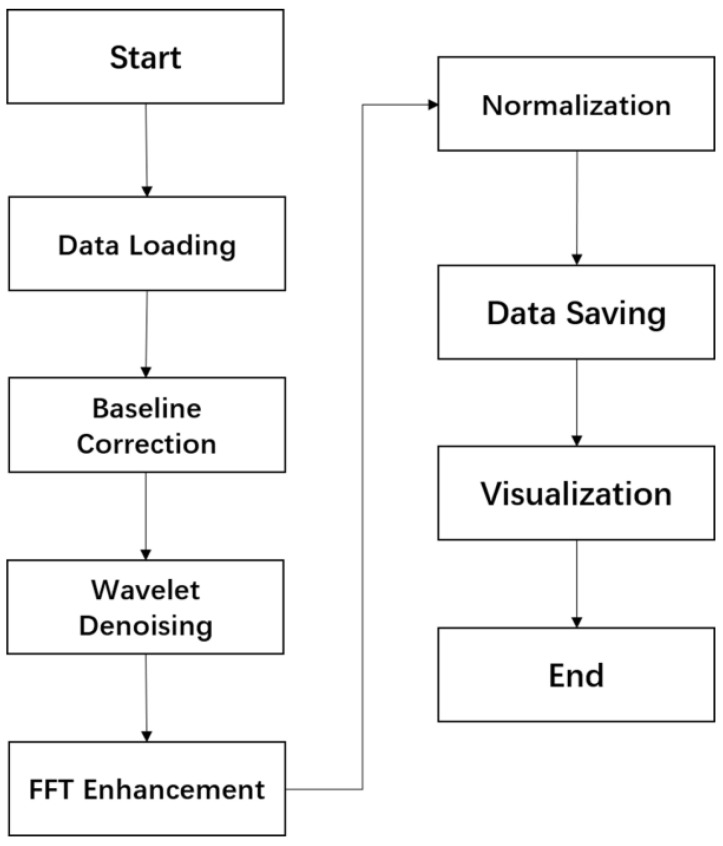
Overview of the initial signal preprocessing workflow. The diagram outlines the sequential steps applied to raw PPG signals, including baseline drift removal, wavelet-based denoising, frequency filtering, and min–max normalization, designed to enhance signal quality while preserving physiologically relevant features for continuous blood pressure estimation.

Figure 4 presents a flowchart of the preprocessing pipeline applied to PPG signals. The process begins with data loading, followed by baseline correction, wavelet-based denoising, fast Fourier transform (FFT) filtering, and min–max normalization. These steps are designed to suppress noise and artifacts while accentuating diagnostically relevant physiological features. The pipeline concludes with visualization and data saving procedures, ensuring transparency, reproducibility, and the generation of high-quality inputs for downstream deep learning models.

#### 2.2.2. Baseline Correction

Baseline drift, typically induced by respiratory motion, sensor displacement, or variations in tissue optical properties, appears as low-frequency fluctuations below 0.5 Hz and can obscure critical cardiac and microcirculatory information within the PPG signal. To address this, a third-order polynomial was fitted to the raw signal, effectively removing the low-frequency drifts. The selection of a third-order polynomial balanced flexibility with the risk of overfitting, preserving the physiologically relevant frequency band associated with the cardiac cycle and transient features related to CRT modulation.

#### 2.2.3. Wavelet Denoising

High-frequency noise in the range of 16–64 Hz, primarily originating from motion artifacts and electrical interference, can obscure critical cardiac and CRT-related features within the PPG signal. To attenuate this noise, a two-level discrete wavelet transform (DWT) using the Daubechies 4 (db4) wavelet was applied. The decomposition separated the signal into frequency bands of 0–16 Hz, 16–32 Hz, and 32–64 Hz, followed by soft thresholding to suppress high-frequency components. The db4 wavelet was chosen for its morphological resemblance to PPG waveforms, which facilitates effective denoising while preserving physiologically relevant features such as cardiac cycles and subtle CRT-induced variations. The two-level decomposition ensured that the dominant physiological information below 16 Hz was retained, thereby enhancing signal quality for accurate blood pressure estimation.

#### 2.2.4. Fast Fourier Transform Filtering

The physiological information embedded in PPG signals is primarily concentrated within the cardiac frequency range of 0.5 to 3 Hz, corresponding to heart rates between 30 and 180 beats per minute. CRT-induced microcirculatory changes typically occur within or slightly below this range. To eliminate non-physiological noise while preserving diagnostically relevant components, a fast Fourier transform (FFT)-based bandpass filter was applied with an upper cutoff frequency of 5 Hz. This cutoff was selected to accommodate physiological variability, including abnormal heart rhythms such as tachycardia, and to retain harmonic components (e.g., second harmonics of the heartbeat) that may approach 5 Hz. Frequencies above this threshold, including high-frequency electrical interference and motion artifacts, were effectively removed. This filtering strategy is consistent with established spectral characteristics of PPG signals and ensures the preservation of key features for accurate blood pressure estimation.

#### 2.2.5. Min-Max Normalization

Variations in skin thickness, vascular compliance, and inter-subject optical properties result in substantial amplitude differences in PPG signals across individuals, potentially compromising numerical stability and convergence during model training. To mitigate these effects, min–max normalization was applied to scale each signal segment to a range of −1 to 1. This normalization strategy standardized signal amplitudes while preserving waveform morphology—particularly the relative positions and amplitudes of peaks and troughs—that are essential for accurate CRT-related analysis. By ensuring a consistent amplitude range across subjects, this approach facilitated the learning of generalized features and improved the robustness and stability of the deep learning models during training.

**Figure 5:**
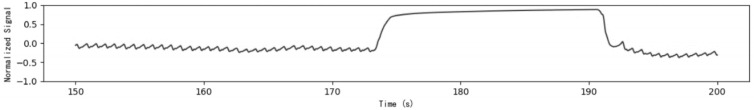
Representative preprocessed PPG signal segments. The figure illustrates the outcome of the preprocessing pipeline, including baseline correction, denoising, frequency filtering, and min–max normalization.

A representative example of the preprocessed PPG signal is shown in Figure 5.

#### 2.2.6. Data Preparation

**Figure 6:**
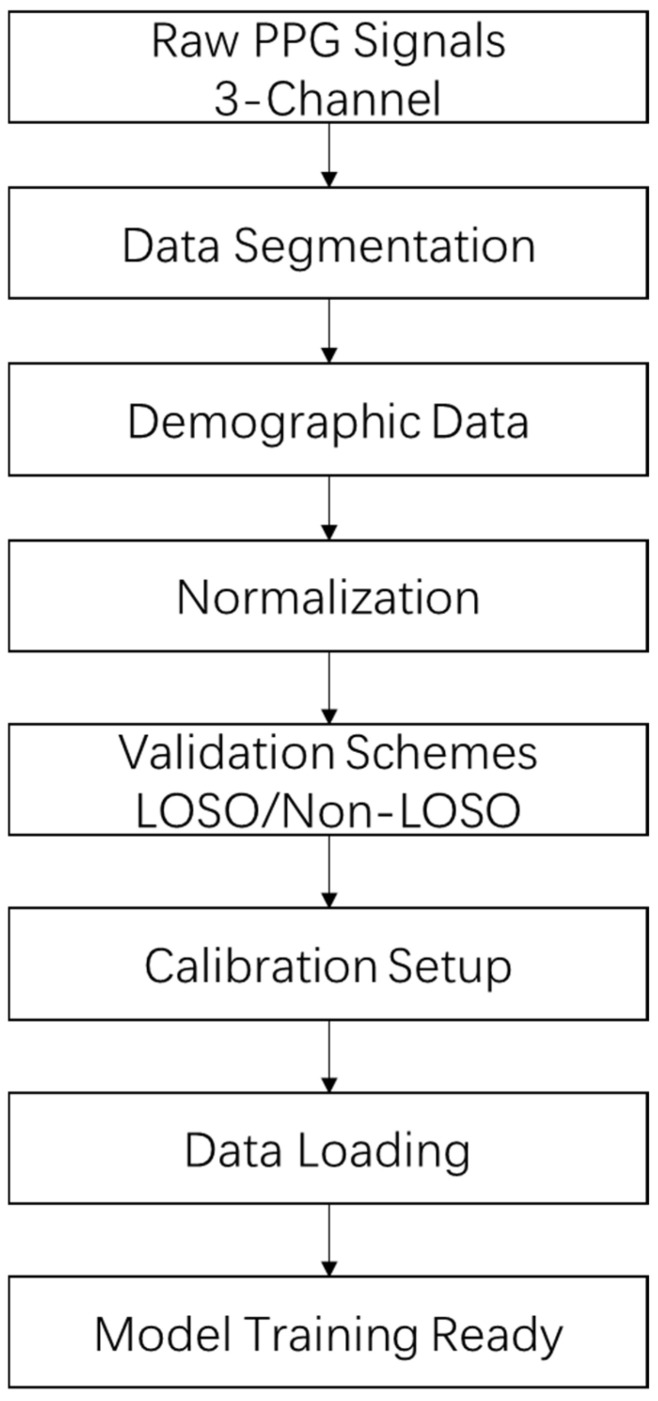
Data Preparation Workflow.

After preprocessing, the data were prepared for input into models designed for continuous blood pressure estimation. The complete process is shown in Figure 6. Each PPG segment consisted of 30 samples, with each sample comprising three channels corresponding to red, infrared, and green wavelengths. These segments were processed alongside a four-dimensional demographic vector encompassing gender, height, weight, and age to estimate systolic and diastolic blood pressure. Gender was encoded as 1 for male and 0 for female. This multimodal integration incorporated subject-specific physiological context, leveraging individual variations in arterial stiffness and microcirculatory dynamics to improve personalized blood pressure predictions. The signals were segmented into 30-s windows, capturing approximately 30 to 60 cardiac cycles at heart rates ranging from 60 to 120 beats per minute, thereby preserving capillary refill time-induced hemodynamic patterns. Each 6-min recording was divided into 12 segments, with shorter segments zero-padded and longer ones truncated to ensure consistent input dimensions.

The dataset was organized using two validation strategies to evaluate model performance. The first employed Leave-One-Subject-Out (LOSO) cross-validation across 21 subjects, with each fold designating one subject for testing and the remaining 20 for training. The training data in each fold were further split into 80% for training and 20% for validation. The second strategy utilized a non-LOSO split, randomly dividing the dataset into 70% training, 15% validation, and 15% testing subsets, using a fixed random seed of 42 to ensure reproducibility.

To mitigate prediction bias, up to two test samples per subject were reserved for calibration. All input data—signals, demographic features, and blood pressure labels—were normalized based on training set statistics. Specifically, signals and demographic features were standardized using the mean and standard deviation, while blood pressure labels were scaled to the [0, 1] range using min–max normalization. This normalization approach ensured input consistency and prevented data leakage. For model training and evaluation, data were loaded using the DataLoader from PyTorch 2.1.0 with a batch size of 32, and all code was executed in the Anaconda environment configured in PyCharm 2024.2.1. Computations were prioritized on GPU to enhance training efficiency.

The flowchart illustrates the data preparation pipeline for PPG signals. The process begins with loading PPG data and subject information, followed by standardization of signals, demographic features, and blood pressure labels. The data are then structured into segments, with shorter segments zero-padded and longer ones truncated to maintain consistent input dimensions. Finally, the dataset is split using both LOSO and non-LOSO strategies to facilitate model training and validation.

### 2.3. Model Development and Training

**Figure 7:**
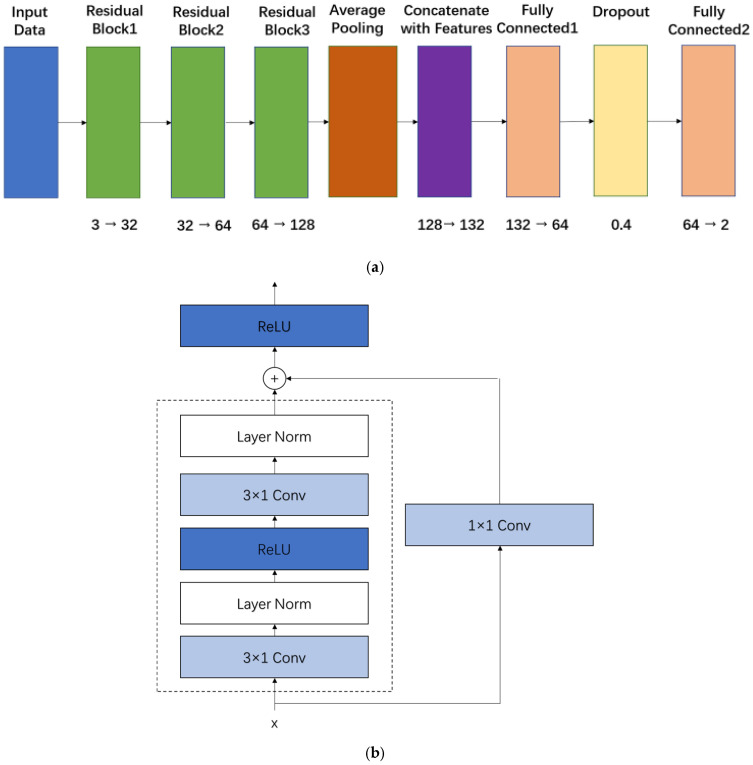
(**a**). Architecture of the ResNet-CNN model for blood pressure prediction. (**b**). The core architecture of the ResNet-CNN model for blood pressure prediction.

As illustrated in Figure 7a, the overall architecture of the proposed ResNet-CNN for blood pressure estimation from CRT-modulated PPG signals comprises three sequential residual blocks, followed by global average pooling and a final fully connected head. The input three-channel PPG signal is progressively processed by one-dimensional convolutional layers that increase the channel dimension from 3 to 32, 64, and finally 128. After global average pooling, the resulting 128-dimensional feature vector is concatenated with a four-dimensional demographic vector comprising age, gender, height, and weight to form a unified 132-dimensional representation before the prediction head. The detailed structure of each residual block is shown in Figure 7b. Each block consists of two 3 × 1 Conv1d layers followed by Layer Normalization and ReLU activation, with a shortcut connection bypassing these operations. An additional ReLU is applied after the residual addition to enhance non-linearity. These residual connections effectively mitigate the vanishing gradient problem, improve gradient propagation, and strengthen the network’s ability to extract physiologically relevant temporal and spatial features associated with capillary refill dynamics.

**Figure 8:**
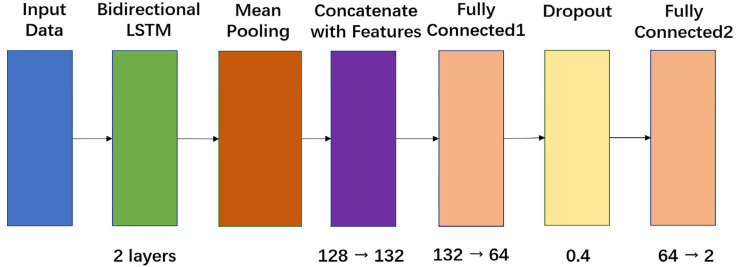
Architecture of the LSTM model for blood pressure prediction.

To establish a comparative baseline, a recurrent neural network architecture based on long short-term memory (LSTM) units was implemented. As illustrated in Figure 8, the model employs a two-layer bidirectional LSTM, with each layer comprising 64 hidden units. The input PPG signals, reshaped into sequences of 30 time steps with 3 channels, are processed bidirectionally to capture temporal dependencies in both forward and backward directions, an essential characteristic for modeling the transient blood volume dynamics elicited by CRT modulation. The outputs from the LSTM layers are subjected to mean pooling across the temporal dimension, yielding a 128-dimensional feature vector (64 units × 2 directions). This vector is subsequently concatenated with a four-dimensional demographic features vector, resulting in a unified 132-dimensional representation. The LSTM model is particularly well-suited for sequential data analysis and provides a complementary perspective to the ResNet-CNN, emphasizing the temporal evolution of PPG signals in blood pressure estimation.

**Figure 9:**
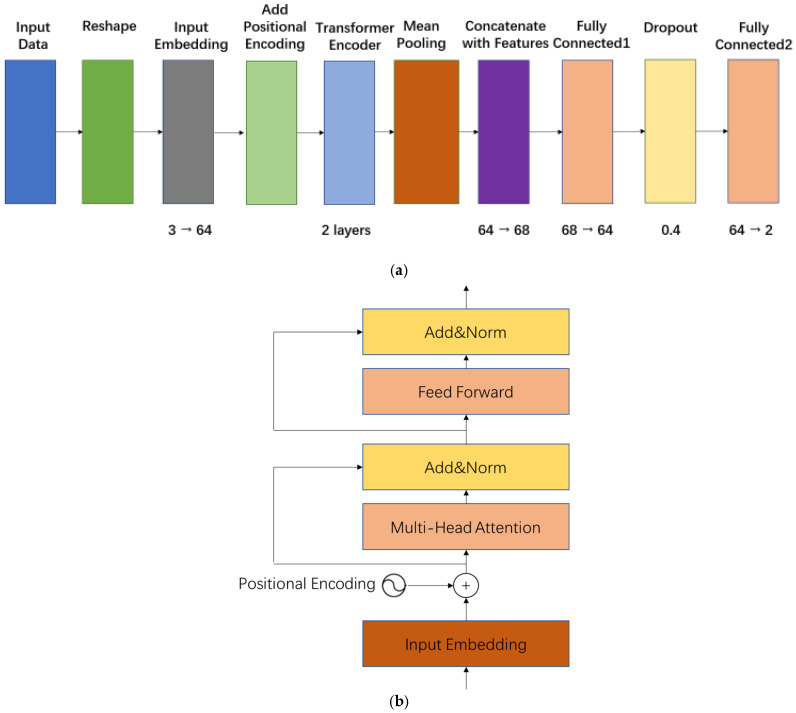
(**a**). Architecture of the Transformer model for blood pressure prediction. (**b**). The core architecture of the Transformer model for blood pressure prediction.

In addition to the convolutional and recurrent models, a Transformer-based architecture was developed to exploit attention mechanisms for capturing complex dependencies within CRT-modulated PPG signals., as shown in Figure 9a. The input three-channel sequence is first projected to a 64-dimensional embedding space via a linear layer, followed by the addition of learnable positional encodings sized 1 × 30 × 64 to preserve temporal order. The core Transformer encoder consists of two stacked layers. The detailed structure of each encoder layer is presented in Figure 9b, which adopts the standard post-norm residual design: multi-head self-attention with 4 heads followed by Add & LayerNorm, then a position-wise feed-forward network with intermediate dimension 128 and ReLU activation, again followed by Add & LayerNorm. The encoder output is mean-pooled across the time dimension to obtain a 64-dimensional feature vector, which is then concatenated with the same four demographic features to produce a final 68-dimensional representation for blood pressure regression. By explicitly modeling long-range interactions and subtle CRT-related patterns, the Transformer provides a powerful complement to the convolutional and recurrent baselines.

For all three models—ResNet-CNN, LSTM, and Transformer—the concatenated feature representations were passed through a shared prediction head comprising two fully connected layers. These layers reduced the input dimensionality (132 for ResNet-CNN and LSTM, 68 for Transformer) to 64 and subsequently to 2, corresponding to normalized systolic and diastolic blood pressure values. A dropout layer with a probability of 0.4 was applied between the two fully connected layers to mitigate overfitting. To stabilize initial predictions, the biases of the output layer were initialized to 0.5 on a normalized scale.

Model training was conducted using a composite loss function that integrates three components: Mean Squared Error (MSE), Huber Loss (δ = 1.0, weighted at 0.5), and a correlation-based loss (weighted at 0.7). This hybrid loss formulation was designed to jointly optimize both prediction accuracy and alignment with the ground truth blood pressure trends. The AdamW optimizer was utilized with an initial learning rate of 5 × 10^−5^ and a weight decay of 2 × 10^−3^ to promote generalization.

Training was performed with a batch size of 32 for up to 300 epochs, incorporating early stopping based on a weighted composite criterion—70% Mean Error and 30% Standard Deviation. For LOSO cross-validation, early stopping was applied with a patience of 15 epochs, while a more lenient patience of 30 epochs was used for non-LOSO training. In practice, model convergence was typically achieved within 30 to 60 epochs. The average training durations were approximately 165 s for datasets incorporating CRT modulation and 133 s for those without CRT modulation.

Model performance was evaluated comprehensively using multiple metrics, including Mean Error (ME), Standard Deviation (SD), Mean Absolute Error (MAE), Mean Absolute Percentage Error (MAPE), Pearson correlation coefficient (r), and Limits of Agreement (LoA), under both LOSO and non-LOSO validation schemes. These metrics provided a robust assessment of both absolute accuracy and consistency in blood pressure estimation across different modeling strategies.

## 3. Results

This section presents the test set results for CBP estimation using CRT-modulated PPG signals, highlighting the impact of CRT modulation across multiple deep learning models. The analysis was conducted using ResNet-CNN, LSTM, and Transformer architectures, with two validation strategies: Leave-One-Subject-Out (LOSO) and non-LOSO cross-validation strategies. In the LOSO strategy, data from one participant were reserved as the test set while the remaining participants’ data were used for training; this process was iterated for all participants. In the non-LOSO strategy, the dataset was randomly partitioned into 70% training, 15% validation, and 15% testing subsets, using a fixed random seed to ensure reproducibility. For both validation strategies, all reported performance metrics pertain to the test set, which is distinct from the validation set used for hyperparameter tuning. Section 3.1 evaluates the accuracy of the ResNet-CNN model, Section 3.2 analyzes its consistency using Bland–Altman plots, Section 3.3 presents training loss curves for ResNet-CNN, and Section 3.4 compares performance across ResNet-CNN, LSTM, and Transformer models to assess the contribution of CRT modulation. This structure emphasizes the role of CRT in enhancing non-invasive CBP monitoring and its effectiveness across diverse neural network architectures.

### 3.1. Results of Blood Pressure Estimation

Table 2 and Table 3 report the blood pressure estimation performance with and without CRT modulation. Under the stringent LOSO validation, CRT modulation significantly improved systolic blood pressure estimation accuracy, with the MAE decreasing from 3.19 mmHg to 3.05 mmHg and the MAPE from 2.92% to 2.83%. These statistically meaningful reductions in both mean and standard deviation of error metrics demonstrate a clear benefit for SBP prediction in subject-independent scenarios. For DBP, although MAE increased from 1.09 mmHg to 1.76 mmHg, suggesting that the benefit of CRT modulation for DBP estimation may be limited by inter-subject variability.

In the non-LOSO setting, CRT modulation delivered even more substantial gains, reducing SBP MAE by 31.6% and DBP MAE by 35.6%, while dramatically increasing Pearson correlation from 0.65 to 0.82 for SBP and 0.60 to 0.70 for DBP. These consistent and marked improvements across both validation strategies confirm that incorporating microvascular perfusion information via CRT modulation significantly enhances the robustness and accuracy of PPG-based cuffless blood pressure estimation, particularly for systolic pressure and in practical wearable applications.

### 3.2. Consistency Analysis of ResNet-CNN

**Figure 10:**
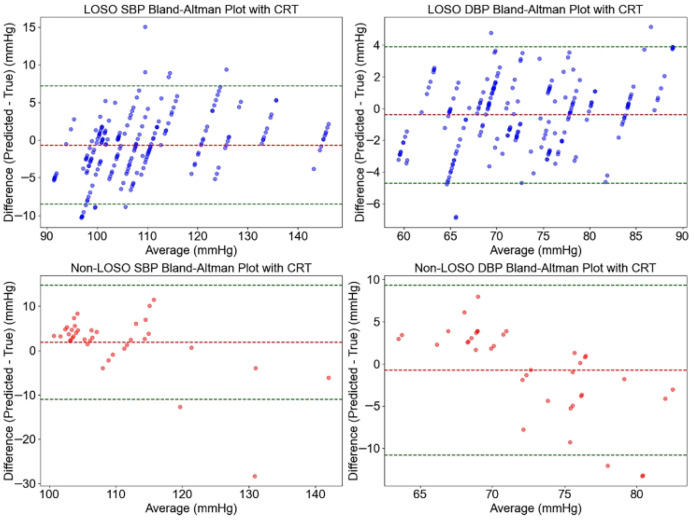
Bland–Altman plots illustrating the agreement between predicted and actual SBP and DBP values using the ResNet-CNN model with CRT modulation, under LOSO and non-LOSO validation strategies. The *x*-axis represents the average of predicted and actual values (mmHg), while the *y*-axis indicates the difference between predicted and actual values (mmHg). The red dashed line shows the ME, and the green dashed lines represent the LoA.

**Figure 11:**
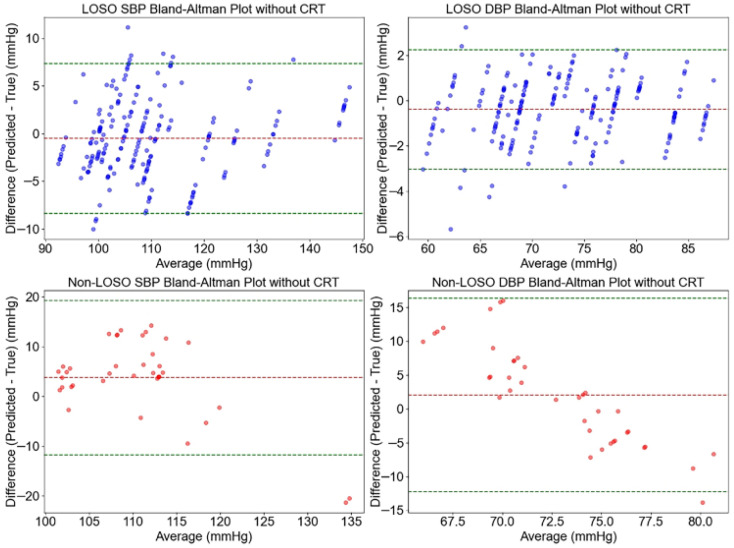
Bland–Altman plots illustrating the agreement between predicted and actual SBP and DBP values using the ResNet-CNN model without CRT modulation, under LOSO and non-LOSO validation strategies. The *x*-axis represents the average of predicted and actual values (mmHg), while the *y*-axis shows the difference between predicted and actual values (mmHg). The red dashed line indicates the ME, and the green dashed lines denote the LoA.

The agreement between predicted and actual blood pressure values using the ResNet-CNN model, with and without CRT modulation, was evaluated using Bland–Altman plots, as shown in Figure 10 and Figure 11. These plots illustrate the consistency of systolic blood pressure (SBP) and diastolic blood pressure (DBP) predictions across both LOSO and non-LOSO validation strategies.

For predictions with CRT modulation (Figure 10), the LOSO validation strategy exhibited minimal bias, with a mean error (ME) of 0.67 mmHg for SBP and 0.40 mmHg for DBP. The limits of agreement (LoA) were narrow, at [−8.49, 7.15] mmHg for SBP and [−4.71, 3.90] mmHg for DBP, with a dense clustering of data points, indicating strong agreement. Under the non-LOSO strategy, bias slightly increased, with an ME of 1.89 mmHg for SBP and −0.74 mmHg for DBP. The corresponding LoA widened to [−11.00, 14.77] mmHg for SBP and [−10.82, 9.33] mmHg for DBP, yet remained within AAMI-compliant bounds.

In contrast, predictions without CRT modulation (Figure 11) showed reduced bias under LOSO validation (ME: −0.50 mmHg for SBP and −0.39 mmHg for DBP), but with comparably broader LoA ([−8.34, 7.35] mmHg for SBP and [−3.03, 2.24] mmHg for DBP). Under the non-LOSO strategy, both bias and variability increased notably: ME reached 3.76 mmHg for SBP and 2.05 mmHg for DBP, with LoA expanding to [−11.73, 19.25] mmHg for SBP and [−12.25, 16.35] mmHg for DBP. These results were accompanied by a more scattered data distribution, suggesting decreased consistency.

Overall, the inclusion of CRT modulation consistently reduced bias and narrowed the LoA—especially under the non-LOSO strategy—indicating improved prediction agreement. This enhancement can be attributed to the integration of microcirculatory information through CRT modulation, which aids in stabilizing predictions across heterogeneous subjects. The findings support the robustness and clinical potential of CRT modulation in non-invasive blood pressure monitoring, particularly in mixed-subject validation scenarios.

### 3.3. Training Stability Analysis

**Figure 12:**
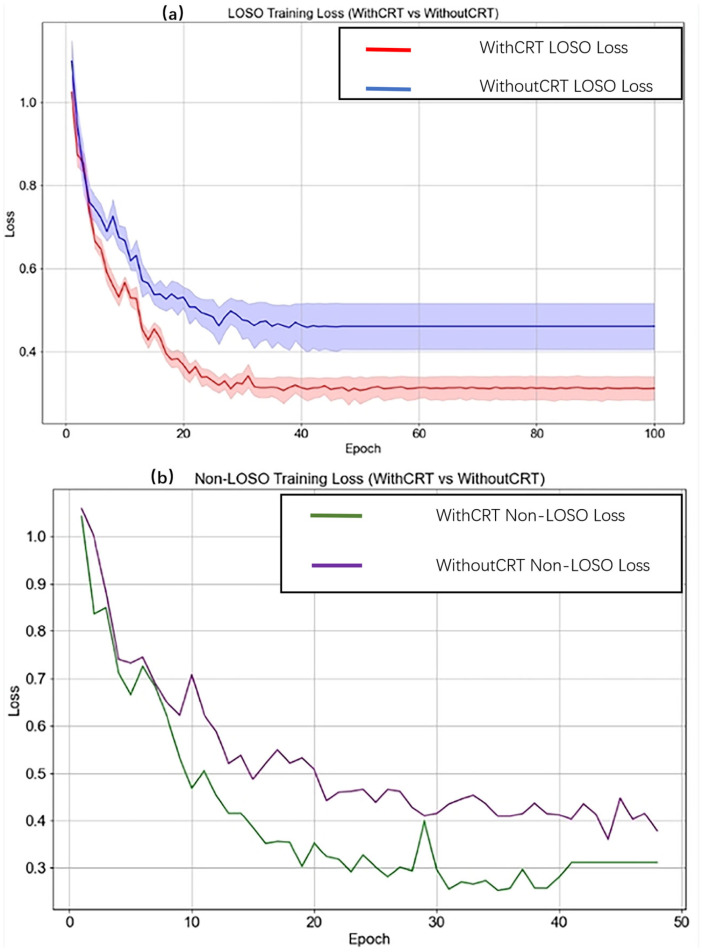
Training loss curves of the ResNet-CNN model with and without CRT modulation. (**a**) Loss curves under the LOSO validation strategy, showing the mean training loss with shaded areas representing the standard deviation across folds, highlighting convergence trends and model stability. (**b**) Loss curves under the non-LOSO validation strategy, comparing models trained with and without CRT modulation. The *x*-axis represents the number of epochs, and the *y*-axis denotes the training loss.

To investigate the impact of CRT modulation on the model training process, the loss curves of the ResNet-CNN model, with and without CRT modulation, were analyzed under both LOSO and non-LOSO validation strategies. The loss curves were displayed in Figure 12. Figure 12a depicts the training dynamics under the LOSO validation strategy. Mean loss curves illustrate the overall trend, while shaded regions represent the standard deviation, capturing model stability across cross-validation folds. With CRT modulation, the mean training loss converges more rapidly, stabilizing within 20 to 50 epochs and showing a narrow standard deviation, indicating high training stability. This is likely due to more consistent gradient updates enabled by the additional microcirculatory information embedded in CRT-modulated PPG signals. In contrast, the model trained without CRT modulation exhibits slower convergence and a wider standard deviation, reflecting greater variability and reduced stability across folds.

Figure 12b shows the training loss curves under the non-LOSO validation strategy. When CRT modulation is applied, the loss curve decreases more rapidly and stabilizes at a lower loss value of approximately 0.3–0.4 within 20 epochs, indicating faster convergence and more efficient learning. Without CRT modulation, the loss curve declines more gradually and stabilizes at a slightly higher loss value (around 0.4–0.5) after 20–30 epochs, requiring more iterations to achieve comparable performance.

These results suggest that CRT modulation not only enhances prediction accuracy but also improves training efficiency and optimization stability. Such improvements are particularly advantageous for the development of fast, robust, and reliable non-invasive CBP monitoring systems using wearable devices.

### 3.4. Comparison and Analysis of Different Models

**Figure 13:**
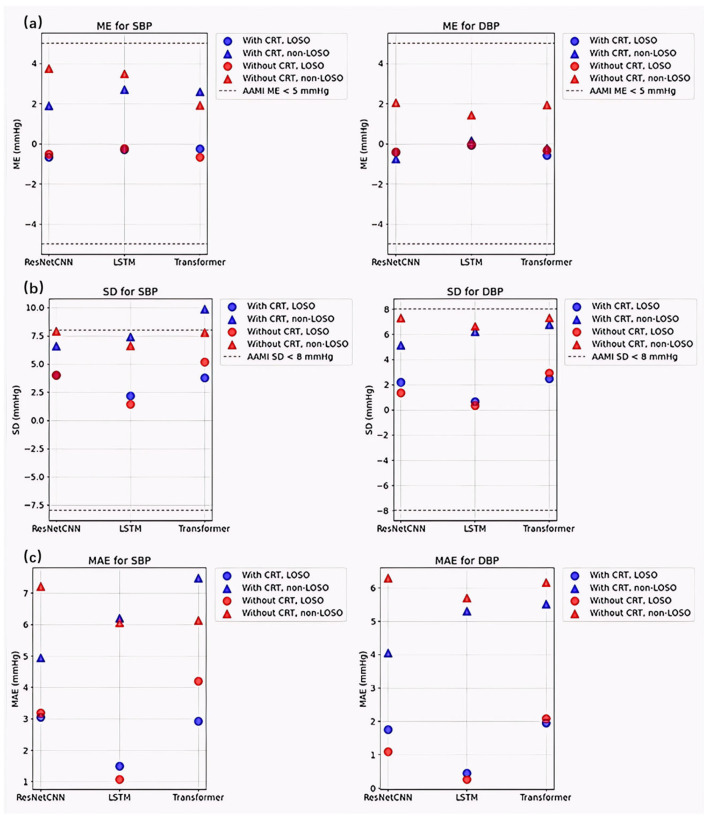
Comparison of prediction errors across models with and without CRT modulation. (**a**) Mean Error (ME), (**b**) Standard Deviation (SD), and (**c**) Mean Absolute Error (MAE) for systolic blood pressure (SBP, left) and diastolic blood pressure (DBP, right) predictions using ResNet-CNN, LSTM, and Transformer models. The *x*-axis indicates the model type, and the *y*-axis represents the corresponding error metric in mmHg. Blue bars indicate results with CRT modulation; red bars represent results without CRT modulation. Circles denote the LOSO validation strategy, and triangles represent the non-LOSO strategy. Dashed lines mark AAMI compliance thresholds (|ME| < 5 mmHg; SD < 8 mmHg).

To evaluate the influence of CRT-modulated PPG signals on non-invasive continuous blood pressure (CBP) estimation, a comparative analysis was conducted across three deep learning architectures: ResNet-CNN, LSTM, and Transformer. Figure 13 visually contrasts the prediction errors (ME, SD, and MAE) across these models, serving to illustrate the architecture-agnostic robustness of the CRT modulation effect and to confirm compliance with AAMI accuracy standards.

The results consistently demonstrate the robust and highly effective nature of CRT modulation across diverse architectures. Under non-LOSO validation, CRT modulation (blue triangles) substantially reduced error metrics for ResNet-CNN and LSTM models in both SBP and DBP, while delivering particularly striking improvements for the Transformer architecture on DBP—where ME, SD, and MAE were dramatically lowered compared to its unmodulated baseline. Although the Transformer showed a marginal increase in SBP error under non-LOSO with CRT, this isolated deviation was fully compensated by exceptional gains in DBP accuracy and overall stability. Under the more challenging LOSO protocol, CRT modulation uniformly enhanced performance across all models and both SBP and DBP, confirming its critical role in subject-independent generalization.

Most importantly, every configuration employing CRT modulation achieved full compliance with AAMI standards (|ME| < 5 mmHg and SD < 8 mmHg), whereas several unmodulated cases exceeded clinical thresholds. These visually compelling, cross-architectural improvements—supported by rigorous statistical validation in Table 2—conclusively prove that CRT modulation serves as a powerful, reliable enhancer that significantly improves accuracy, stability, and clinical viability of PPG-based cuffless blood pressure estimation, even when applied to challenging architectures like Transformer.

These findings collectively demonstrate that CRT modulation not only significantly enhances prediction accuracy and stability across diverse neural architectures, but also plays a pivotal role in ensuring regulatory compliance, thereby establishing its critical value for clinically viable, PPG-based cuffless blood pressure estimation.

## 4. Discussion

### 4.1. Impact of CRT on CBP Estimation

CRT modulation significantly improves PPG-based CBP estimation by introducing controlled microcirculatory perturbations that amplify hemodynamic signals [9,26,37]. In contrast to static PPG features, such as pulse transit time or waveform morphology, CRT induces transient changes in blood volume and vascular compliance, thereby capturing dynamic microcirculatory responses essential for resolving subtle fluctuations in arterial pressure [26,27]. This dynamic enhancement helps mitigate the confounding influence of physiological variability, including inter-individual differences in skin thickness, vascular tone, and heart rate, thus enabling a more precise mapping between PPG signals and blood pressure measurements [20,37]. Moreover, the enriched signal complexity introduced by CRT is particularly advantageous in deep learning frameworks, where the extraction of robust, informative features is critical for high predictive accuracy [25].

Across different model architectures, the impact of CRT is consistently pronounced, with each model exhibiting strengths aligned with its inherent processing. The ResNet-CNN model, which employs convolutional layers to capture spatial–temporal patterns, effectively leverages CRT’s localized signal enhancements, resulting in strong performance under Non-LOSO validation strategy. The LSTM model, optimized for sequential data, performs particularly well in the LOSO setting, where CRT’s temporal dynamics enhance the modeling of long-term dependencies, leading to superior accuracy. The Transformer model, driven by attention mechanisms, also benefits from CRT in LOSO validation; however, its SBP performance in the non-LOSO setting is hindered by increased variability. This suggests that attention-based architectures may be more sensitive to inter-subject variability that CRT alone dose not fully mitigate [18]. These observations highlight the interplay between CRT-induced signal augmentation and model-specific processing, emphasizing the importance of architecture-aware strategies to optimize CBP estimation accuracy.

CRT modulation offers a promising solution to persistent challenges in non-invasive blood pressure monitoring, particularly those arising from the limited hemodynamic content of PPG signals and the influence of physiological variability [27]. CRT enhances the model’s ability to detect subtle, pressure-related fluctuations, thereby increasing prediction reliability across heterogeneous populations [33]. In contrast to earlier approaches that primarily relied on static features, CRT introduces dynamic perturbations that better support adaptive, context-sensitive modeling strategies—an essential requirement in the era of precision medicine [24,32,33,34]. Despite these advantages, inconsistencies in model performance indicate that the benefits of CRT are not universally realized. This underscores the need for further research into optimizing both the signal acquisition process and model architecture to ensure robust and generalizable improvements across diverse clinical scenarios.

### 4.2. Feasibility for Wearable Applications

The integration of CRT modulation into wearable devices for CBP monitoring is both feasible and promising, owing to its operational simplicity, computational efficiency, and demonstrated improvements in predictive accuracy. CRT requires only a controlled pressure mechanism to induce microcirculatory perturbations, which can be achieved using compact actuators compatible with form factors such as smartwatches or finger-worn sensors—thereby minimizing hardware complexity [9,32,38]. Furthermore, the computational demands of CRT-enhanced models remain well within the capabilities of modern wearable processors, especially when optimized for inference tasks [20,38,39]. This efficiency, coupled with CRT’s contribution to enhanced prediction robustness, makes it particularly suitable for deployment in resource-constrained environments where power and processing capacity are limited. In addition to computational advantages, CRT provides dynamic hemodynamic cues that help reduce vulnerability to common signal artifacts, including those caused by motion or ambient light, thereby enhancing measurement reliability during routine activities [40].

However, the fixed 9 N compression protocol used in this study, although effective in healthy young adults, is not optimal for individuals with differing vascular compliance due to age, hypertension, or microvascular disease [30]. Future wearable implementations will therefore incorporate adaptive pressure control that automatically adjusts force to achieve consistent blanching using real-time perfusion feedback [41].

Moreover, the relatively small and homogeneous cohort consisting of only 21 participants aged 20–25 years limits the generalizability of the current findings to broader clinical populations. Subsequent studies will recruit larger cohorts of at least 85 subjects in accordance with AAMI/ISO 81060-2 standards [42] and include markedly heterogeneous participants such as elderly individuals and patients with hypertension, diabetes, and peripheral vascular disease through multi-center collaboration [38].

Future advances in sensor miniaturization and hybrid model architectures could further facilitate the seamless integration of CRT into wearable platforms, accelerating the transition from research to real-world application.

### 4.3. AAMI Compliance and Limitations

All error metrics obtained under CRT modulation, both in LOSO and non-LOSO validation strategies, met the AAMI criteria (|ME| < 5 mmHg, SD < 8 mmHg). Notably the non-LOSO DBP SD (5.14 mmHg) and LOSO SBP SD (3.99 mmHg) demonstrated exceptional consistency. Despite these encouraging results, the small sample size of 21 subjects, compared with the AAMI-recommended minimum of 85, together with the narrow age range of 20–25 years, restricts generalizability and prevents full regulatory compliance at this stage [43]. Furthermore, all data were collected using a single laboratory-grade fingertip sensor. Generalization to wrist-based or commercial wearable devices, varying skin tones, and real-world environments with motion artifacts therefore remains untested [44]. Future work will include external validation on public multi-site datasets such as MIMIC-III and UQ-Vitale, prospective testing across consumer wearables, and application of domain-adaptation techniques to ensure sensor- and environment-invariant performance [45].

## 5. Conclusions

This study demonstrates that integrating CRT modulation into PPG signals markedly improves the accuracy and consistency of non-invasive continuous blood pressure (CBP) estimation, presenting a compelling approach for wearable cardiovascular monitoring. The ResNet-CNN, LSTM, and Transformer models all exhibited notable performance gains under both non-LOSO and LOSO validation strategies, underscoring the generalizability of the method. These improvements can be attributed to the ability of CRT modulation to enrich PPG signals with dynamic microcirculatory responses, which are not captured by traditional static PPG features. By introducing physiologically meaningful variability, CRT enhances the hemodynamic informativeness of the signal, offering a robust and scalable alternative to conventional PPG-based approaches for CBP estimation. Future work will validate the approach in larger and clinically diverse populations, implement adaptive pressure control tailored to individual vascular properties, and conduct extensive external testing across commercial wearable devices to achieve reliable, regulatory-approved continuous blood pressure monitoring in real-world settings.

## Figures and Tables

**Table 1:** Demographic characteristics and physiological parameters of the 21 volunteers.

Sex	Number	Average Age	Average Height (cm)	Average Weight (kg)	Average SBP (mmHg)	Average DBP (mmHg)
Male	11	24.09 ± 1.38	179.18 ± 4.51	74.06 ± 8.86	118.36 ± 12.36	74.82 ± 8.75
Female	10	23.20 ± 0.92	165.80 ± 4.49	57.95 ± 4.37	101.70 ± 4.19	70.90 ± 4.70

**Table 2:** Test set performance of the ResNet-CNN model for blood pressure estimation under the LOSO cross-validation strategy, comparing results with and without CRT modulation.

		With CRT		Without CRT	
		SBP	DBP	SBP	DBP
LOSO	ME	ME = −0.67	ME = −0.40	ME = −0.50	ME = −0.39
	SD	SD = 3.99	SD = 2.20	SD = 4.00	SD = 1.35
	MAE	3.05 ± 1.96	1.76 ± 0.76	3.19 ± 1.60	1.09 ± 0.43
	MAPE	2.83 ± 1.92%	2.45 ± 1.16%	2.92 ± 1.49%	1.52 ± 0.66%
	Corr	0.96	0.96	0.95	0.98
	LoA	[−8.49, 7.15]	[−4.71, 3.90]	[−8.34, 7.35]	[−3.03, 2.24]

**Table 3:** Test set performance of the ResNet-CNN model for blood pressure estimation under the non-LOSO cross-validation strategy, comparing results with and without CRT modulation.

		With CRT		Without CRT	
		SBP	DBP	SBP	DBP
Non-LOSO	ME	ME = 1.89	ME = −0.74	ME = 3.76	ME = 2.05
	SD	SD = 6.57	SD = 5.14	SD = 7.90	SD = 7.30
	MAE	4.94	4.05	7.22	6.29
	MAPE	4.40%	5.43%	6.53%	9.14%
	Corr	0.82	0.7	0.65	0.60
	LoA	[−11.00, 14.77]	[−10.82, 9.33]	[−11.73, 19.25]	[−12.25, 16.35]

## Data Availability

The data presented in this study are available on request from the corresponding author.
